# Extremely Low Microsatellite Diversity but Distinct Population Structure in a Long-Lived Threatened Species, the Australian Lungfish *Neoceratodus forsteri* (Dipnoi)

**DOI:** 10.1371/journal.pone.0121858

**Published:** 2015-04-08

**Authors:** Jane M. Hughes, Daniel J. Schmidt, Joel A. Huey, Kathryn M. Real, Thomas Espinoza, Andrew McDougall, Peter K. Kind, Steven Brooks, David T. Roberts

**Affiliations:** 1 Australian Rivers Institute, Griffith University, Brisbane, Queensland, Australia; 2 Terrestrial Zoology and Molecular Systematics Unit, Western Australian Museum, Welshpool, Western Australia, Australia; 3 Department of Natural Resources and Mines, Bundaberg, Queensland, Australia; 4 Queensland Department of Agriculture, Fisheries and Forestry, Brisbane, Queensland, Australia; 5 Seqwater, Ipswich, Queensland, Australia; Tuscia University, ITALY

## Abstract

The Australian lungfish is a unique living representative of an ancient dipnoan lineage, listed as ‘vulnerable’ to extinction under Australia’s *Environment Protection and Biodiversity Conservation Act 1999*. Historical accounts indicate this species occurred naturally in two adjacent river systems in Australia, the Burnett and Mary. Current day populations in other rivers are thought to have arisen by translocation from these source populations. Early genetic work detected very little variation and so had limited power to answer questions relevant for management including how genetic variation is partitioned within and among sub-populations. In this study, we use newly developed microsatellite markers to examine samples from the Burnett and Mary Rivers, as well as from two populations thought to be of translocated origin, Brisbane and North Pine. We test whether there is significant genetic structure among and within river drainages; assign putatively translocated populations to potential source populations; and estimate effective population sizes. Eleven polymorphic microsatellite loci genotyped in 218 individuals gave an average within-population heterozygosity of 0.39 which is low relative to other threatened taxa and for freshwater fishes in general. Based on *F*_ST_ values (average over loci = 0.11) and STRUCTURE analyses, we identify three distinct populations in the natural range, one in the Burnett and two distinct populations in the Mary. These analyses also support the hypothesis that the Mary River is the likely source of translocated populations in the Brisbane and North Pine rivers, which agrees with historical published records of a translocation event giving rise to these populations. We were unable to obtain bounded estimates of effective population size, as we have too few genotype combinations, although point estimates were low, ranging from 29 - 129. We recommend that, in order to preserve any local adaptation in the three distinct populations that they be managed separately.

## Introduction

For species of conservation concern (i.e. endangered or vulnerable), increasing or maintaining effective population size (*N*_e_) and thus maintaining levels of genetic diversity has been considered critically important [1]. This is important because species with low levels of genetic diversity may be less resilient to environmental stressors such as pathogens [2] and climate change [3]. Various management options have been suggested for populations with low genetic diversity, including ‘genetic rescue’ through the addition of individuals from other populations in an attempt to maintain or increase genetic diversity in threatened populations [4]. In some cases, the introduction of a very small number of individuals has produced significant fitness benefits [5]. However, there is also the possibility of reduced fitness if the introduced individuals are genetically differentiated from the resident population [4]. Thus, management of threatened species requires information on both the genetic diversity within populations and levels of genetic divergence between populations.

Managing long-lived species requires an understanding of the factors affecting population dynamics which can be difficult to determine from current day adult populations as the historical drivers for the present day structure may have been different when they were influencing key recruitment processes in the past [6,7]. However, an analysis of the genetic structure of present day populations can help understand past patterns of historical demographic change, such as changes in population size and also evidence of inbreeding or non-random mating patterns [1]. A number of recent studies of long-lived species have suggested that long generation times can ameliorate the loss of genetic variation due to small effective population sizes [7,8].

**Fig 1:**
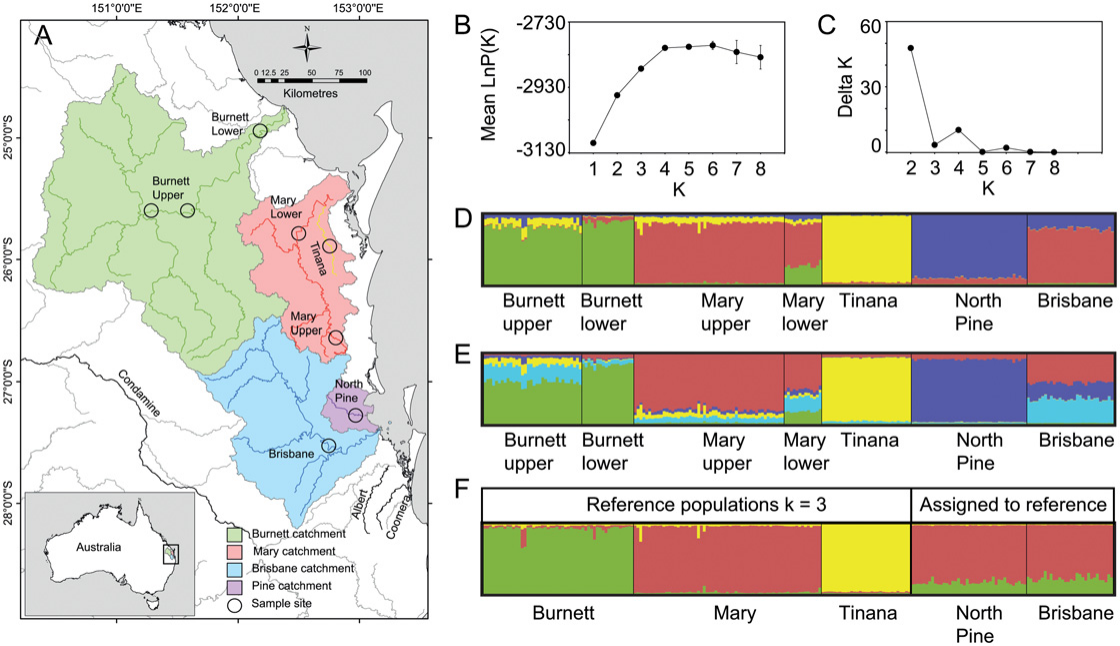
Sample sites and clustering analysis of multilocus microsatellite data performed using STRUCTURE software. (**A)** Map of southeast Queensland highlighting sampled catchments and the seven sampling locations. (**B**) Plot of values for the mean likelihood of each genetic cluster (*K*) for *K* = 1 to 8, where the error bar represents one standard deviation. (**C**) Plot of Delta *K* calculated as the mean of the second-order rate of change in likelihood of *K* divided by the standard deviation of the likelihood of *K*. (**D**) Bar plot of estimated membership of each individual in *K* = 4 clusters. Black bars separate the seven population sample groups. (**E**) Bar plot of estimated membership of each individual in *K* = 5 clusters. (**F**) Genetic assignment of translocated population samples to three reference genetic groups (*K* = 3) representing three genetically distinct natural populations. Three reference groups comprise individuals from Burnett River, Mary River and Tinana Creek. Unknown groups assigned to the reference comprise individuals from North Pine River and Brisbane River.

The Australian lungfish *Neoceratodus forsteri* is the most primitive member of the subclass Dipnoi, and the only extant representative of the group in Australia [9]. Australian lungfish reach large sizes (up to 150 cm and 20 kg) and are very long-lived, with some individuals known to live for over 80 years [9,10]. The species also has a very long evolutionary history, having existed with little change in morphology for around 100 million years since the early Cretaceous [9,11]. The Australian lungfish was historically widespread in drainages of eastern Australia before its range contracted to a small area of southeastern Queenlsand during the Pleistocene [12]. The species is currently only found in a few rivers in southeast Queensland, although it is thought to occur naturally in only two river systems, the Mary River and the Burnett River [9,13] see Fig 1A. Populations in other rivers (Brisbane, North Pine, Condamine, Coomera, Albert, see Fig 1A), are thought to be the result of translocations, some of which were documented in the late nineteenth century [14]. Recruitment of juveniles into the population has rarely been observed since the discovery of the lungfish in 1870, leading to the belief that the population may be at risk of extinction [15] and that translocation was an option to increase the distribution and spread the risk across multiple river systems [16]. Lungfish are quite abundant in parts of the Brisbane River (David Roberts, pers comm.), with fewer in the North Pine River and fewer individuals in the other rivers to which they were translocated. Populations are all dominated by adults, with little evidence of recent or regular recruitment in most of these populations [17]. It has been declared a nationally vulnerable species under the Environment Protection and Biodiversity Conservation Act 1999 and is listed as a no-take species under Schedule 2 of the Fisheries Regulation 2008 under the Fisheries Act 1999 (Queensland). For the purposes of managing lungfish populations, Fisheries Queensland has always assumed that Burnett and Mary populations are natural and that the other populations have resulted from translocation (Peter Kind, pers comm.).

Genetic differences between sub-populations can occur when they become geographically isolated by barriers which can include physical barriers such as water falls or disconnected rivers, or chemical barriers such as salinity gradients. *N*. *forsteri* are not tolerant of elevated salinities [9] and therefore it is likely that populations in different coastal drainages are effectively isolated from one another by salinity barriers. Depending on how long the populations have been isolated, this is likely to have resulted in genetic differentiation, although the rate that this differentiation will have accrued is likely to be much slower than for other freshwater fish species, due to the very long generation time of lungfish, not reaching maturity until about 20 years of age [9].

A previous genetic study examined 24 allozyme loci and a large portion of mitochondrial DNA and reported extremely low levels of genetic variation [13]. Only two allozyme loci were polymorphic, each with only two alleles, and most individuals from the Mary, Burnett and Brisbane Rivers shared a single mitochondrial haplotype [13]. The authors concluded that the very low genetic variation was probably the result of past bottlenecks resulting from periodic or extended dry periods during the Pleistocene, but they were unable to draw definite conclusions about the levels of genetic structure between river systems, as the very low levels of genetic variation meant that there was low statistical power. The authors also suggested the possibility of inbreeding in the natural populations, detecting significant heterozygote deficits in three of four comparisons.

Microsatellites, due to their high mutation rate and the fact that most are non-coding and therefore not subject to selection [18] are likely to provide more power to test these questions. Huey *et al*. [19] recently developed a panel of novel polymorphic microsatellite loci for the lungfish. In this study, we use these microsatellites to test the following hypotheses:

There will be significant genetic structure between the two ‘natural populations’, the Burnett River and the Mary River, because they are separate drainage basins, isolated by the ocean and the lungfish is an obligate freshwater fish; there will be low levels of genetic differentiation between putatively translocated populations in the Brisbane River and North Pine River and at least one of the natural populations (the assumed source) because the translocations occurred within the last 150 years and the generation time for the lungfish is very long, thus slowing the possibility of genetic divergence since translocation. We also use the genetic data to attempt to estimate effective population sizes, as previous work has suggested that these may be very low and thus represent a risk for the future of the species.

## Methods

### Sampling Strategy

Tissue and scale samples were collected from 218 individuals from seven localities in the Burnett, Mary, Brisbane and North Pine River catchments (Fig 1A). Two localities were sampled from the Burnett (Upper Burnett and Lower Burnett) and three from the Mary (Upper Mary, Lower Mary and Tinana Creek). Lungfish collected from multiple positions within the same tributary were pooled together for each of these sites. A single locality was sampled from each of the Brisbane (below Wivenhoe Dam) and North Pine Rivers (below North Pine Dam). The number of individuals from each location is shown in Table 1.

**Table 1:** Summary of genetic variation in populations of *Neoceratodus forsteri* based on eleven microsatellite loci.

	*N*	*H*_O_	*H*_E_	*F*_IS_	*AR*	*AR*_priv_	*N*_E_	*Bottleneck*	*M-ratio*
Burnett_upper	34	0.360 ± 0.192	0.395 ± 0.177	0.070*	2.42	0.06	29 (8, ∞)	NS	0.53
Burnett_lower	18	0.307 ± 0.176	0.372 ± 0.204	0.186*	2.27	0.21	∞ (8, ∞)	NS	0.55
Mary_upper	52	0.368 ± 0.393	0.393 ± 0.185	0.065*	2.22	0.02	129 (28, ∞)	NS	0.54
Mary_lower	13	0.471 ± 0.287	0.421 ± 0.196	-0.107	2.12	0.00	∞ (20, ∞)	NS	0.58
Tinana	31	0.370 ± 0.145	0.433 ± 0.168	0.130*	2.18	0.09	58 (12, ∞)	NS	0.52
North Pine	40	0.347 ± 0.171	0.349 ± 0.169	0.006	2.10	0.13	49 (14, ∞)	NS	0.52
Brisbane	30	0.373 ± 0.252	0.361 ± 0.234	-0.034	2.14	0.04	∞ (19, ∞)	NS	0.55

Number of specimens (*N*); observed heterozygosity (*H*_O_) and expected heterozygosity (*H*_E_) presented as average across loci with one standard deviation; inbreeding coefficient (*F*_IS_) averaged across loci with asterisk denoting populations with significant heterozygote deficiency. Effective population size (*N*_E_) estimates with 95% parametric confidence intervals in parentheses were calculated using the linkage disequilibrium method excluding alleles with frequency <0.05. Allelic richness (*AR*) and private allelic richness (*AR*_priv_) rarefied to minimum sample size of 16 alleles. Bottleneck results correspond to one-tail Wilcoxon test for heterozygote excess, NS = not significant. M-ratio is the Garza-Williamson index following [28].

### Ethics Statement

All field and experimental protocols carried out in this study were approved by the Griffith University Animal Ethics Committee. All procedures were carried out according to Australian Ethics Committee protocol numbers CA2011/10/551 (Seqwater) and ENV/17/14/AEC (Griffith University).

### Laboratory Methods

A standard phenol-chloroform extraction method [20] was used to extract DNA from scales or fin clip tissue samples. Of 336 microsatellite primers developed and tested on *N*. *forsteri*, 115 successfully amplified a product of expected size and of these, 94 were monomorphic and 21 were variable [19]. Further testing of the 21 variable loci reported by Huey *et al*. [19], showed nine had inconsistent amplification results and were excluded from this study, while the remaining 11 loci were screened across all individuals. The 11 loci include: 1LF041, 2LF034, 2LF026, 1LF005, 2LF049, 2LF069, 2LF041, 1LF044, 2LF031, 2LF032 and 1LF117 [19]. PCR amplification methods can be found in Huey *et al*. [19]. Microsatellites were fluorescently labeled using the multi-tailed primer tagging method of Real *et al*. [21] and run on an ABI3130 Fragment Analyser using the G5 dye set. Alleles were scored using GeneMapper V3.1 (Applied Biosystems).

### Data Analyses

#### Genetic diversity and effective population size

Genetic diversity averaged across eleven loci within each of the seven population samples was quantified from observed (*H*_O_) and expected (*H*_E_) heterozygosity using ARLEQUIN v3.5.1.2 [22]. Measures of genetic diversity standardized for sample size including allelic richness (*AR*) and private allelic richness (*AR*_priv_) were calculated using HP-Rare [23]. Polymorphism information content (PIC) was evaluated for each locus using CERVUS v3.0.7 [24], where PIC > 0.5 is considered highly informative; 0.5 > PIC > 0.25 reasonably informative; and PIC<0.25 slightly informative [25]. Tests for deviation from Hardy-Weinberg Equilibrium (HWE) for each locus-population combination were carried out using exact tests implemented in ARLEQUIN. The inbreeding coefficient (*F*_IS_) was estimated over all loci for each population using the Analysis of Molecular Variance (AMOVA) framework in ARLEQUIN and tested for deviation from the random expectation by 10,000 permutations of alleles among individuals within populations.

Genetic evidence for a recent reduction in local population size was tested using the software BOTTLENECK v1.2.02 [26] which compares observed and expected heterozygosity where the expected value is the equilibrium expectation conditioned on the observed number of alleles [27]. The Two-Phased (TPM) mutation model was used, incorporating 95% single-step changes with variance of multiple-step changes set to 12%. Statistical significance was evaluated from 5000 simulations using the one-tailed Wilcoxon sign-rank test as recommended from the power analysis of Piry et al. [26]. We also calculated Garza and Williamson’s [28] M-ratio for each population sample using ARLEQUIN. The M-ratio is the mean ratio of the number of alleles to the range of allele size. It is sensitive to population bottlenecks because it measures the proportion of unoccupied allelic states given the range in allele size, and this ratio is reduced as alleles are randomly lost due to drift [28].

An estimate of effective population size (*N*_e_) was made for each of the seven population samples using the single-sample linkage disequilibrium method [29], implemented in the software NeEstimator v2.01 [30]. Low frequency alleles (<0.05) were excluded from analysis and parametric estimates of 95% confidence intervals were reported.

#### Population genetic structure

Genetic structure among the seven population samples was quantified by estimating pair-wise and global *F*_ST_ values as a weighted average over eleven loci in ARLEQUIN. These were tested for significant deviation from panmictic expectations by 10,000 permutations of individuals among populations. The critical value (α) was corrected for multiple tests using the BY False Discovery Rate method (BY-FDR) which controls experiment-wide Type I error without the loss of power associated with the Bonferroni adjustment [31]. A hierarchical AMOVA was also run, with catchment as the highest level of the hierarchy. The grouping was Burnett (upper and lower), Mary (upper, lower and Tinana) Pine and Brisbane. The AMOVA was performed in ARLEQUIN as a weighted average over the 11 loci with significance of F-statistics determined by 1,000 permutations.

We tested for the existence of distinct genetic groups within the pooled set of 218 individual multi-locus genotypes using model-based Bayesian clustering. The probability of an admixture model was tested for up to eight clusters (*K*) using STRUCTURE 2.3.1 [32]. The location of individuals in sampling groups was incorporated as a prior in the analysis (LOCPRIOR model). This model is useful in cases where genetic structure is weak and the model does not tend to over specify structure where none exists [33]. Models were tested using 20 independent MCMC simulations, each consisting of 2×10^6^ iterations after a burn-in of 2×10^5^ iterations. The most likely number of clusters was assessed first by plotting the mean probability of data for successive values of *K* to find a plateau in probability values and then choosing the smallest of these values [34]. Secondly, we calculated Delta *K* or the second-order rate of change L˝ (*K*) following Evanno *et al*. [35] using the online application Structure Harvester [36]. We also used STRUCTURE to assign individuals from putatively translocated population samples (Brisbane and North Pine) to the major genetic clusters formed among the known natural/source populations (Burnett, Mary, Tinana). This analysis utilized the USEPOPINFO model, setting the natural samples to POPFLAG = 1, and translocated samples to POPFLAG = 0. Allele frequencies were updated using only individuals with POPFLAG = 1 so that individuals from translocated samples were forced to cluster with one or more of the natural population groups. Twenty replicate STRUCTURE runs were collated using the software CLUMPP [37] and visualized with DISTRUCT [38].

## Results

Levels of diversity were extremely low whether viewed as an average over loci within population samples (Table 1), or as individual loci across samples (Table 2). Expected heterozygosity of the 11 polymorphic loci averaged 0.39 across the seven sample sites and allelic richness per site averaged just over 2 when sample sizes were standardized across populations at 16 individuals (Table 1). In terms of individual loci, the number of alleles per locus averaged 3.55 and the average PIC was 0.35 with only two loci (1LF005 and 1LF044) achieving PIC >0.5 which is considered a highly informative level of variation (Table 2).

**Table 2:** Locus-by-locus summary of genetic variation across seven population samples of *Neoceratodus forsteri*.

Locus name	N_A_	*H*_O_	*H*_E_	PIC	*F*_ST_
1LF041	2	0.284	0.295	0.251	0.073 (<0.0001)
2LF034	3	0.342	0.438	0.35	0.106 (<0.0001)
2LF026	3	0.442	0.553	0.454	0.213 (<0.0001)
1LF005	6	0.427	0.602	0.527	0.243 (<0.0001)
2LF049	2	0.479	0.487	0.368	0.043 (<0.01)
2LF069	3	0.213	0.206	0.186	0.039 (<0.01)
2LF041	4	0.526	0.568	0.479	0.070 (<0.0001)
1LF044	4	0.545	0.61	0.546	0.102 (<0.0001)
2LF031	3	0.428	0.483	0.381	0.049 (<0.01)
2LF032	3	0.053	0.052	0.051	0.019 (<0.05)
1LF117	6	0.134	0.203	0.198	0.038 (<0.01)
mean	3.55	0.352	0.409	0.345	

Number of alleles (N_A_); observed heterozygosity (*H*_O_) and expected heterozygosity (*H*_E_); Polymorphism information content (PIC); fixation index (*F*_ST_), P-values in parentheses based on 10,000 permutations.

Four of the five sample sites from natural populations had significant deficits of heterozygotes, with only the site with a small sample size (Mary lower) not showing a significant deficit (Table 1). In contrast, both sample sites from assumed translocated populations (North Pine and Brisbane) had *F*_IS_ values very close to zero. The estimates of *N*_*E*_ were mostly quite small, but confidence levels were broad, indicating that the magnitude of sampling error was comparable to the signal of among locus genetic linkage in the data. This was probably because of the low levels of diversity overall, limiting the power to detect linkage disequilibrium. All bottleneck tests were non-significant and M-ratio values ranged from 0.5–0.6 for each population sample (Table 2).

The overall *F*_ST_ value across 11 loci was 0.111 and was highly significant (P<0.001). Each of the 11 locus-by-locus *F*_ST_ values were also significant at P<0.05 and ranged from 0.019 for locus 2LF032 to 0.243 for locus 1LF005 (Table 2). Most of the pairwise *F*_ST_ values between the seven population samples were significant (Table 3) and ranged from 0.015 to 0.197. Only three comparisons involving the lower Mary, which had a much smaller sample size than other sites, were non-significant. In the hierarchical AMOVA, the *F*_CT_ value was 0.033 (P>0.05), the *F*_SC_ value was 0.085 (P<0.001), indicating significant differences within at least one of the catchments. This result was possibly caused by the highly significant differences between Tinana Creek and the other two Mary River sites.

**Table 3:** Pairwise estimates of *F*_ST_ for eleven polymorphic microsatellite loci.

	Burnett_upper	Burnett_lower	Mary_upper	Mary_lower	Tinana	North Pine
Burnett_upper	-					
Burnett_lower	**0.067**	-				
Mary_upper	**0.067**	**0.038**	-			
Mary_lower	**0.036**	0.028	0.015	-		
Tinana	**0.082**	**0.155**	**0.087**	**0.105**	-	
North Pine	**0.149**	**0.149**	**0.102**	**0.108**	**0.197**	**-**
Brisbane	**0.100**	**0.060**	**0.031**	0.023	**0.142**	**0.068**

Comparisons in bold were significantly differentiated after adjusting the critical value using the FDR B-Y correction (α = 0.5; adjusted critical value = 0.01928).

Examination of likelihood scores produced by STRUCTURE from 20 replicate runs across *K* values from one to eight showed the mean likelihood score (Ln(*K*)) plateaued at *K* = 4 while the Delta K method indicated *K* = 2, although the latter method did not show relatively strong support for any value (Fig 1B and 1C). We infer that *K* = 4 captures the majority of structure in our sample and the admixture bar plot for *K* = 4 (Fig 1D) shows a strong relationship between the population samples and four genetic groups. Clustering the data using values of *K* > 4 did not produce biologically sensible solutions but merely forced some populations to accommodate more admixture (see Burnett and Brisbane for *K* = 5, Fig 1E). We had hypothesized that the populations in each of the river systems would constitute separate populations. This was partly supported, in that the upper Mary sample was distinct from the upper and lower Burnett samples (Fig 1D). The lower Mary was distinct from the upper Burnett, but not from the lower Burnett. Furthermore, unexpectedly, the sample from Tinana Creek, part of the Mary River catchment, was very distinct from any of the samples from other natural populations (Fig 1D). Also, unexpectedly, the sample from the North Pine River was distinct from all natural populations (Fig 1D). The Brisbane River sample was most similar to the Mary upper and lower samples (Fig 1D). In summary, there is evidence for three genetically distinct populations within the native range of Australian lungfish, two in the Mary catchment and one in the Burnett. A fourth distinct group was found in a putatively translocated population, North Pine River.

Assignment of putatively translocated samples (North Pine and Brisbane) to potential source populations in the native range of lungfish (Burnett, Mary) using STRUCTURE was complicated by strong genetic structure within the Mary catchment where the sample from Tinana Creek comprised a distinct genetic group. For this reason we set potential source populations in the native range of lungfish to *K* = 3 groups using the USEPOPINFO model in STRUCTURE. These three groups are geographically distinct and correspond with 1) Burnett River (pooled Burnett upper and Burnett lower samples); 2) Mary river (pooled Mary upper and Mary lower samples); 3) Tinana Creek. The data from putatively translocated samples (North Pine and Brisbane) were treated as having unknown affinity and assigned to the three genetic clusters from the native range. Twenty replicate runs of STRUCTURE at *K* = 3, showed all individuals sampled from putatively translocated populations (North Pine and Brisbane) had a close affinity to the Mary River genetic cluster (Fig 1F).

## Discussion

### Genetic Diversity

The level of genetic variation in this long-lived vulnerable species is indeed extremely low. This had already been reported for allozymes by Frentiu *et al*. [13], who found only 2 polymorphic allozyme loci out of 24 sampled. They also found very little variation in mitochondrial DNA, spanning both the control region and part of the ATPase 6 and 8 genes. During screening of microsatellite markers for this project, only 19% of 115 clearly interpretable loci were polymorphic. Recent reviews of microsatellite development using comparable next-generation sequencing methods suggest an average return of around 60% polymorphic loci is typical across a broad taxonomic range of species [39,40]. While the level of microsatellite polymorphism in the Australian lungfish is therefore unusually low, it is higher than reported for the endangered Hawaiian monk Seal *Monachus schauinslandi* of 5% from a survey of 154 loci [1]. The monk seal is considered depauperate in genetic diversity (mean heterozygosity of polymorphic loci = 0.49) owing to a severe population bottleneck due to human exploitation [1]. Our results for the Australian lungfish suggest an even lower average heterozygosity of 0.39 for polymorphic microsatellite loci. This value is also well below the mean of 0.68 for a survey of freshwater fishes by McCusker and Bentzen [41]. This value is also well below the expected heterozygosity reported for other large-bodied, long-lived and threatened riverine fishes including the arapaima, *Arapaima gigas* [42], the beluga sturgeon, *Huso huso* [43] and Murray cod, *Maccullochella peelii* [44]. However, heterozygosity values lower than reported here for lungfish are known in freshwater fishes. For example, an average *H*_E_ of 0.18 was recorded in marble trout populations subject to periodic catastrophic floods causing genetic bottlenecks [45].

We surveyed a range of populations from the species’ native range as well as putatively translocated populations but found very similar levels of diversity in terms of heterozygosity and allelic richness across all sites. Therefore low genetic diversity does not appear to be a consequence of recent human activities as in the case of the monk seal. The Australian lungfish has either experienced a number of bottlenecks in the past, possibly due to Pleistocene range contraction as suggested by Frentiu *et al*. [13] and Kemp [12], or has had a naturally very low effective population size. The low levels of allozyme and mitochondrial DNA diversity [13] and microsatellite diversity reported here, even with the extremely long life-time, suggest that effective population sizes have been low for a significant period.

There is also some genetic evidence of inbreeding in the natural populations, as all but one of them had significant heterozygote deficits. This was also observed in the two allozyme loci analysed by Frentiu *et al*. [13], so it is likely to be an accurate reflection of mating patterns. It is very interesting that this heterozygote deficit was not observed in either of the translocated populations. This is particularly interesting, as Kemp [46] reported deformities in lungfish embryos sampled from the Brisbane River population, which could potentially be evidence of inbreeding depression, but our genetic data do not support a significant deviation from random mating as a likely explanation for this observation.

While our microsatellite markers were sufficiently variable for an analysis of population structure, they were not sufficiently variable for good estimates of *N*_e_. The linkage disequilibrium method works on the number of allelic combinations and so when variability is low, precision is severely affected. Waples *et al*. [29] showed that when the number of alleles per locus is around 2 (as is the case here), you would need to screen 180 loci to obtain the same precision as for a typical microsatellite data set with 20 microsatellite loci and 10 alleles per locus. Given the low levels of microsatellite variation in this species, it will be necessary to develop a SNP analysis, where it should be possible to identify many more loci, which will not be any less variable than our current microsatellite loci.

### Genetic Differentiation

We had hypothesized that samples taken from populations believed to be natural would be differentiated between the two river systems, because the species is restricted to freshwater and highly intolerant of elevated salinities. This prediction was partly supported in that samples from the Burnett and the main stem of the Mary River were differentiated and appeared as distinct populations in the STRUCTURE analysis. However, we had not expected that the sample from Tinana Creek, a tributary of the Mary River, would also be distinct. Clearly there is a barrier separating Tinana Creek from the rest of the Mary River system despite their close proximity to one another (Fig 1A). Tinana Creek flows into the Mary River not far from the mouth, with both localities having tidal estuarine reaches in the lower sections. Salinities in these lower sections will be higher than tolerable limits during normal river flow conditions, thus restricting migration between the two systems. However, these localities flood periodically, reducing salinities throughout the system and therefore it might be expected that fish could disperse between the two systems at such times. It appears however that this does not happen or occurs very rarely. Earlier genetic studies [13] did not uncover this divergent population because Tinana Creek was not included in their analysis.

Interestingly, a number of other freshwater species show genetic differentiation between Tinana Creek and the rest of the Mary River. These include the threatened Mary River cod *Maccullochella mariensis* [47], the rainbowfish *Melanotaenia dublayi* [48] and a freshwater crayfish *Cherax dispar* [49]. The fact that a number of species show a similar pattern suggests that this divergence is caused by the same process, probably historical isolation.

The other unexpected finding was that the sample from the North Pine River was differentiated from all the samples taken from natural populations and from the Brisbane River population, particularly in the initial STRUCTURE analysis (Fig 1D). This difference could have two explanations. First, there could have been a natural population in the North Pine River historically. If this population had been in the river over many generations then the differentiation would be expected. However, this seems unlikely, as there have been no reports of lungfish in this river system before the translocations [50]. Given the proximity to Brisbane it is likely that a natural population would have been discovered by early settlers and explorers pre1896. An alternative explanation is that the differentiation reflects a founder effect resulting from the very small number of individuals introduced. Although five fish were initially taken to the North Pine river, Kemp [50] reports that O’Connor, who was responsible for the translocation, later reported that only three survived the journey. Artificial introductions of small numbers of individuals can result in significant genetic differentiation between the source population and the translocated one [51]. If this was the case, then we might have expected to see a significant result from the bottleneck tests for this sample. However, as the diversity was so low in the source population, possibly the BOTTLENECK test, which relies on an elevated heterozygosity relative to the number of alleles expected under mutation drift equilibrium [27], had limited power. A recent simulation study by Peery *et al*. [52] showed that overall, the power of this bottleneck test to detect even quite large population declines can be quite low unless sample sizes are very large (around 100) and there are a large number of loci (around 20). In contrast, M-ratio results were indicative of a reduction in lungfish population size in all our samples, as values were below the critical value of 0.68, which is derived from putatively stable wild populations by Garza and Williamson [28]. However Peery *et al*. [52] also found power to detect bottlenecks using this method was low when genetic diversity is low, which is the case with lungfish. Deciding among possible explanations for the unexpected genetic differentiation of the North Pine River lungfish sample is not possible based on our microsatellite data, and we defer a final conclusion until a more extensive genomic data set is available.

Our results for the Brisbane River sample support the hypothesis that this population has come from the Mary River proper. It clearly has not come from Tinana Creek, or from the Burnett. This finding is consistent with historical records that the source population of various translocations made by O’Connor in the late nineteenth century was the Mary River [14]. Some literature suggests that the Brisbane River population may in fact not be the result of a translocation [50,53]. In fact Kemp and Hynen [53] identified jaw bones collected from the Brisbane River, dating back to 3850 ybp as belonging to lungfish. However, results from Frentiu’s study, while not totally convincing due to the limited variability of the markers used, found no evidence for the Brisbane River population being distinct [13]. Our results also find no evidence to support this notion, as individuals sampled from the Brisbane River do not form a unique genetic cluster but group together with individuals from the Mary River (upper and lower). When the Brisbane sample was treated as unknown and classified against the three known natural population groups (Burnett, Mary, Tinana), it was most similar to the Mary group. Furthermore comparisons between the Brisbane River and Mary River samples based on *F*_ST_ values were lower than with other samples.

Evidence for genetic distinction between Mary and Brisbane river lungfish populations was found using randomly amplified DNA fingerprinting (RAF) markers in an unpublished report by Lissone [54]. This data showed a single band (out of ~1,400 total bands) at high frequency in the Brisbane sample that was absent in other samples. This result could indicate that Brisbane is a distinct natural population but it is also consistent with a founder event associated with the documented historical translocation. In addition, the well-known repeatability issues associated with PCR-based DNA fingerprinting dictates cautious interpretation of these results [55]. Lissone’s [54] data therefore does not confirm that lungfish present in the Brisbane river today represent a natural and distinct population as asserted by Kemp and Huynen [53]. However it is plausible that a natural population did occur in the Brisbane historically as supported by the recent finding of ancient jawbones [53]. Again, to finally resolve this issue will require a more extensive analysis of a wide portion of the lungfish genome.

We conclude that there are at least three distinct natural populations of the Australian lungfish, the Mary River proper, Tinana Creek and the Burnett River. Our evidence suggests that the North Pine River and Brisbane River populations have been sourced from the Mary River proper. While numbers of lungfish may be quite high in some of these sites, the extremely low levels of genetic diversity at neutral loci might be cause for concern, as they may also reflect low diversity in coding genes that are responsible for the long-term survival of the populations. If the effective population sizes are indeed very low, then this diversity could be further eroded over the next few generations. Given that these microsatellite loci did not have sufficient power to estimate effective population size with any precision, we propose to use a RAD-sequencing approach [56] to sample a much higher proportion of the genome and therefore make use of whatever variation we can detect.

‘Genetic rescue’ has sometimes been proposed as a strategy to increase genetic diversity in small and/or threatened populations [4]. However, in the present case, further movement of individuals between the three natural populations is not recommended. Furthermore, it is recommended that the three natural populations be managed as such. Our analysis indicates that each is a discrete population, so mixing them may inhibit any local adaptation [4]. The two translocated populations have similar levels of genetic diversity to the natural populations and do not show evidence of inbreeding or genetic bottlenecks, although a SNP analysis with many more loci may be more powerful to detect these effects. Nevertheless, it appears that these populations contain as much diversity as the natural populations and may provide a source of individuals and genetic diversity should any of the other populations experience extreme declines.

## Supporting Information

S1 Microsatellite Dataset Microsatellite dataset used for all analyses in Excel spreadsheet format. Data for each individual in rows, data for each locus in columns. Data for each allele of each locus is recorded in a separate column with the header row giving the name of the locus. Missing data marked by “?”. First column is individual sample code; second column is population code corresponding to population groupings analysed in the manuscript.
(XLSX)

## References

[1] Schultz JK, Baker JD, Toonen RJ, Bowen BW (2009) Extremely Low Genetic Diversity in the Endangered Hawaiian Monk Seal (*Monachus schauinslandi*). Journal of Heredity 100: 25–33. 10.1093/jhered/esn077 18815116

[2] Hawley DM, Sydenstricker KV, Kollias GV, Dhondt AA (2005) Genetic diversity predicts pathogen resistance and cell-mediated immunocompetence in house finches. Biology Letters 1: 326–329. 17148199 10.1098/rsbl.2005.0303 PMC1617150

[3] Allendorf FW, Luikart GH, Aitken SN (2012) Conservation and the genetics of populations. West Sussex UK: Wiley-Blackwell 624 p.

[4] Tallmon DA, Luikart G, Waples RS (2004) The alluring simplicity and complex reality of genetic rescue. Trends in Ecology & Evolution 19: 489–496. 16701312 10.1016/j.tree.2004.07.003

[5] Ingvarsson PK (2001) Restoration of genetic variation lost—The genetic rescue hypothesis. Trends in Ecology & Evolution 16: 62–63. 11165697 10.1016/s0169-5347(00)02065-6

[6] Kuo CH, Janzen FJ (2004) Genetic effects of a persistent bottleneck on a natural population of ornate box turtles (*Terrapene ornata*). Conservation Genetics 5: 425–437.

[7] Lippe C, Dumont P, Bernatchez L (2006) High genetic diversity and no inbreeding in the endangered copper redhorse, *Moxostoma hubbsi* (Catostomidae, Pisces): the positive sides of a long generation time. Molecular Ecology 15: 1769–1780. 16689897 10.1111/j.1365-294X.2006.02902.x

[8] Goossens B, Chikhi L, Jalil MF, Ancrenaz M, Lackman-Ancrenaz I, Mohamed M, et al (2005) Patterns of genetic diversity and migration in increasingly fragmented and declining orang-utan (*Pongo pygmaeus*) populations from Sabah, Malaysia. Molecular Ecology 14: 441–456. 15660936 10.1111/j.1365-294X.2004.02421.x

[9] Pusey BJ, Kennard M, Arthington A (2004) Freshwater fishes of north-eastern Australia. Collingwood, VIC: CSIRO Publishing 684 p.

[10] James KM, Fallon SJ, McDougall A, Espinoza T, Broadfoot C (2010) Assessing the potential for radiocarbon dating the scales of Australian lungfish (*Neoceratodus forsteri*). Radiocarbon 52: 1084–1089.

[11] Kemp A, Molnar R (1981) *Neoceratodus forsteri* from the Lower Cretaceous of New South Wales, Australia. Journal of Paleontology 55: 211–217.

[12] Kemp A (1997) A revision of Australian Mesozoic and Cenozoic lungfish of the family neoceratodontidae (Osteichthyes:Dipnoi), with a description of four new species. Journal of Paleontology 71: 713–733.

[13] Frentiu FD, Ovenden JR, Street R (2001) Australian lungfish (*Neoceratodus forsteri*: Dipnoi) have low genetic variation at allozyme and mitochondrial DNA loci: a conservation alert? Conservation Genetics 2: 63–67.

[14] O'Connor D (1897) Report on preservation of Ceratodus. Proceedings of the Royal Society of Queensland 10–14: 101–102.

[15] Illidge T (1893) On *Ceratodus forsteri* . Proceedings of the Royal Society of Queensland 10: 40–44.

[16] Kemp A (1986) The biology of the Australian lungfish, *Neoceratodus forsteri* (Krefft 1870). Journal of Morphology: 181–198. 3959085

[17] Arthington AH (2009) Australian lungfish, Neoceratodus forsteri, threatened by a new dam. Environmental Biology of Fishes 84: 211–221.

[18] Jarne P, Lagoda PJL (1996) Microsatellites, from molecules to populations and back. Trends in Ecology & Evolution 11: 424–429. 21237902 10.1016/0169-5347(96)10049-5

[19] Huey JA, Real KM, Mather PB, Chand V, Roberts DT, Espinoza T, et al (2013) Isolation and characterization of 21 polymorphic microsatellite loci in the iconic Australian lungfish, *Neoceratodus forsteri*, using the Ion Torrent next-generation sequencing platform. Conservation Genetics Resources 5: 737–740.

[20] Sambrook J, Fritsch EF, Maniatis T (1989) Molecular Cloning: A Laboratory Manual. New York: Cold Spring Harbor Laboratory Press 9.14–19.23 p.

[21] Real KM, Schmidt DJ, Hughes JM (2009) *Mogurnda adspersa* microsatellite markers: multiplexing and multi-tailed primer tagging. Conservation Genetics Resources 1: 411–414.

[22] Excoffier L, Lischer H (2010) Arlequin suite ver 3.5: a new series of programs to perform population genetics analyses under Linux and Windows. Molecular ecology resources 10: 564–567. 10.1111/j.1755-0998.2010.02847.x 21565059

[23] Kalinowski ST (2005) HP-RARE 1.0: a computer program for performing rarefaction on measures of allelic richness. Molecular Ecology Notes 5: 187–189.

[24] Kalinowski ST, Taper ML, Marshall TC (2007) Revising how the computer program CERVUS accommodates genotyping error increases success in paternity assignment. Molecular Ecology 16: 1099–1106. 17305863 10.1111/j.1365-294X.2007.03089.x

[25] Botstein D, White RL, Skolnick M, Davis RW (1980) Construction of a Genetic-Linkage Map in Man Using Restriction Fragment Length Polymorphisms. American Journal of Human Genetics 32: 314–331. 6247908 PMC1686077

[26] Piry S, Luikart G, Cornuet JM (1999) BOTTLENECK: A computer program for detecting recent reductions in the effective population size using allele frequency data. Journal of Heredity 90: 502–503.

[27] Cornuet JM, Luikart G (1996) Description and power analysis of two tests for detecting recent population bottlenecks from allele frequency data. Genetics 144: 2001–2014. 8978083 10.1093/genetics/144.4.2001 PMC1207747

[28] Garza JC, Williamson EG (2001) Detection of reduction in population size using data from microsatellite loci. Molecular Ecology 10: 305–318. 11298947 10.1046/j.1365-294x.2001.01190.x

[29] Waples RS, Do C (2008) LDNE: a program for estimating effective population size from data on linkage disequilibrium. Molecular Ecology Resources 8: 753–756. 10.1111/j.1755-0998.2007.02061.x 21585883

[30] Do C, Waples RS, Peel D, Macbeth GM, Tillett BJ, Ovenden JR (2014) NEESTIMATOR v2: re-implementation of software for the estimation of contemporary effective population size (N-e) from genetic data. Molecular Ecology Resources 14: 209–214. 10.1111/1755-0998.12157 23992227

[31] Narum SR (2006) Beyond Bonferroni: Less conservative analyses for conservation genetics. Conservation Genetics 7: 783–787.

[32] Pritchard JK, Stephens M, Donnelly P (2000) Inference of population structure using multilocus genotype data. Genetics 155: 945–959. 10835412 10.1093/genetics/155.2.945 PMC1461096

[33] Hubisz MJ, Falush D, Stephens M, Pritchard JK (2009) Inferring weak population structure with the assistance of sample group information. Molecular Ecology Resources 9: 1322–1332. 10.1111/j.1755-0998.2009.02591.x 21564903 PMC3518025

[34] Pritchard JK, Wen X, Falush D (2010) Documentation for structure software: Version 2.3.

[35] Evanno G, Regnaut S, Goudet J (2005) Detecting the number of clusters of individuals using the software STRUCTURE: a simulation study. Molecular Ecology 14: 2611–2620. 15969739 10.1111/j.1365-294X.2005.02553.x

[36] Earl DA, Vonholdt BM (2012) STRUCTURE HARVESTER: a website and program for visualizing STRUCTURE output and implementing the Evanno method. Conservation Genetics Resources 4: 359–361.

[37] Jakobsson M, Rosenberg NA (2007) CLUMPP: a cluster matching and permutation program for dealing with label switching and multimodality in analysis of population structure. Bioinformatics 23: 1801–1806. 17485429 10.1093/bioinformatics/btm233

[38] Rosenberg NA (2004) DISTRUCT: a program for the graphical display of population structure. Molecular Ecology Notes 4: 137–138.

[39] Gardner MG, Fitch AJ, Bertozzi T, Lowe AJ (2011) Rise of the machines—recommendations for ecologists when using next generation sequencing for microsatellite development. Molecular Ecology Resources 11: 1093–1101. 10.1111/j.1755-0998.2011.03037.x 21679314

[40] Schoebel CN, Brodbeck S, Buehler D, Cornejo C, Gajurel J, Hartikainen H, et al (2013) Lessons learned from microsatellite development for nonmodel organisms using 454 pyrosequencing. Journal of Evolutionary Biology 26: 600–611. 10.1111/jeb.12077 23331991

[41] McCusker MR, Bentzen P (2010) Positive relationships between genetic diversity and abundance in fishes. Molecular Ecology 19: 4852–4862. 10.1111/j.1365-294X.2010.04822.x 20849560

[42] Araripe J, do Rego PS, Queiroz H, Sampaio I, Schneider H (2013) Dispersal Capacity and Genetic Structure of *Arapaima gigas* on Different Geographic Scales Using Microsatellite Markers. Plos One 8. 10.1371/journal.pone.0054470 PMC3553164 23372730

[43] Dudu A, Georgescu SE, Burcea A, Florescu I, Costache M (2014) Analysis of genetic diversity in beluga sturgeon, *Huso huso* (Linnaeus, 1758) from the Lower Danube River using DNA markers. Scientific Papers Animal Science and Biotechnologies 47: 64–68.

[44] Rourke ML, McPartlan HC, Ingram BA, Taylor AC (2010) Biogeography and life history ameliorate the potentially negative genetic effects of stocking on Murray cod (*Maccullochella peelii peelii*). Marine and Freshwater Research 61: 918–927.

[45] Pujolar JM, Vincenzi S, Zane L, Jesensek D, De Leo GA, Crivelli AJ (2011) The Effect of Recurrent Floods on Genetic Composition of Marble Trout Populations. Plos One 6. 10.1371/journal.pone.0023822 PMC3169565 21931617

[46] Kemp A (2014) Abnormal development in embryos and hatchlings of the Australian lungfish, *Neoceratodus forsteri*, from two reservoirs in south-east Queensland. Australian Journal of Zoology 62: 63–79.

[47] Huey JA, Espinoza T, Hughes JM (2013) Natural and anthropogenic drivers of genetic structure and low genetic variation in the endangered freshwater cod, *Maccullochella mariensis* . Conservation Genetics 14: 997–1008.

[48] Lee L (2013) Multi scale genetic structure of crimson spotted rainbowfish (Melanotaenia duboulayi) with reference to urban environmental restoration.: Griffith University 70 p.

[49] Bentley AI, Schmidt DJ, Hughes JM (2010) Extensive intraspecific genetic diversity of a freshwater crayfish in a biodiversity hotspot. Freshwater Biology 55: 1861–1873.

[50] Kemp A (2012) The world of lungfish: a personal perspective: Anne Kemp. 298 p.

[51] Carvalho GR, Shaw PW, Hauser L, Seghers BH, Magurran AE (1996) Artificial introductions, evolutionary change and population differentiation in Trinidadian guppies (*Poecilia reticulata*:Poeciliidae). Biological Journal of the Linnean Society 57: 219–234.

[52] Peery MZ, Kirby R, Reid BN, Stoelting R, Doucet-Beer E, Robinson S, et al (2012) Reliability of genetic bottleneck tests for detecting recent population declines. Molecular Ecology 21: 3403–3418. 10.1111/j.1365-294X.2012.05635.x 22646281

[53] Kemp A, Huynen L (2014) Occurrence of lungfish in the Brisbane River, Queensland, Australia dates back to 3850 yr BP. Journal of Archaeological Science 52: 184–188.

[54] Lissone I (2003) Conservation genetics and the Australian lungfish *Neoceratodus forsteri* (Krefft 1870); a spatio-temporal study of population structure. [Master of Science Thesis]: University of the Sunshine Coast.

[55] Pérez T, Albornoz J, Domínguez A (1998) An evaluation of RAPD fragment reproducibility and nature. Molecular Ecology 7: 1347–1357. 9787445 10.1046/j.1365-294x.1998.00484.x

[56] Baird NA, Etter PD, Atwood TS, Currey MC, Shiver AL, Lewis ZA, et al (2008) Rapid SNP Discovery and Genetic Mapping Using Sequenced RAD Markers. Plos One 3. 10.1371/journal.pone.0003376 PMC2557064 18852878

